# Xylose–glucose co-fermentation to ethanol by *Escherichia coli* strain MS04 using single- and two-stage continuous cultures under micro-aerated conditions

**DOI:** 10.1186/s12934-019-1191-0

**Published:** 2019-08-23

**Authors:** Marco T. Fernández-Sandoval, Juvencio Galíndez-Mayer, Francisco Bolívar, Guillermo Gosset, Octavio T. Ramírez, Alfredo Martinez

**Affiliations:** 1 Departamento de Ingeniería Celular y Biocatálisis, Instituto de Biotecnología, Universidad Nacional Autónoma de México, Av. Universidad 2001, Colonia Chamilpa, 62210 Cuernavaca, Morelos Mexico; 2 Laboratorio de Bioingeniería, Escuela Nacional de Ciencias Biológicas, Instituto Politécnico Nacional, Carpio y Plan de Ayala, Col. Santo Tomás, CP 11340 Mexico, D.F., Mexico; 3 Departamento de Medicina Molecular y Bioprocesos, Instituto de Biotecnología, Universidad Nacional Autónoma de México, Av. Universidad 2001, Colonia Chamilpa, 62210 Cuernavaca, Morelos Mexico

**Keywords:** Ethanol, Continuous culture, Simultaneous co-fermentation, *Escherichia coli*, Xylose, Glucose, Serial bioreactors, Volumetric ethanol productivity

## Abstract

**Background:**

Simultaneous co-fermentation of mixed sugars is an important feature to consider in the production of ethanol from lignocellulosic biomass hydrolysates because it enhances the overall ethanol yield and volumetric productivity during fermentation. Continuous cultures can be used during ethanol production from lignocellulosic hydrolysates to prevent catabolite repression by glucose on other sugars, such as xylose, and thus promote the simultaneous and total consumption of sugars and reduce fermentation time. The use of single- and two-stage continuous cultures under micro-aerated conditions for simultaneous consumption of xylose and glucose, and fermentation to ethanol by ethanologenic *Escherichia coli* strain MS04 was studied. Mineral medium supplemented with glucose, xylose and sodium acetate, was used to compare continuous cultures performance to batch cultures.

**Results:**

Single-stage continuous cultures under micro-aerated conditions allowed the total co-consumption of a mixture of glucose and xylose (7.5 and 42.5 g/L, respectively) in mineral medium, with steady state ethanol production of 18 g/L, and a volumetric ethanol productivity of 0.9 g/L h, when low dilution rates (0.05 h^−1^) were used. However, the volumetric productivity was lower than the batch process under similar conditions (1.3 g/L h). Conversely, micro-aerated two-stage continuous culture enhanced the volumetric productivity up to 1.6 g/L h at a dilution rate of 0.15 h^−1^, with a total consumption of sugars and a slight reduction of the overall ethanol yield.

**Conclusions:**

The total and simultaneous consumption of glucose and xylose by the ethanologenic *E. coli* strain MS04 was accomplished by using two-stage continuous culture under micro-aerated conditions with an increase in the volumetric ethanol productivity of 23% and 78% when compared to batch and single-stage continuous cultures, respectively. Multi-stage continuous cultivation can be used to promote the simultaneous consumption of all sugars contained in biomass hydrolysates, and thus increase the volumetric ethanol productivity of the fermentation process.

## Background

Ethanol is the foremost biotechnological commodity produced worldwide, and the most widely used biofuel nowadays, [1–3]. Also, ethanol combustion is cleaner than gasoline and more efficient, since it increases octane levels, helping to reduce the air pollutant emissions and carbon dioxide net emissions, because of better oxidation of hydrocarbons [3–5]. Ethanol is currently produced from different food crops in the so-called first-generation bioethanol (FGB) [3, 6], but, due to several drawbacks with the use of such substrates, second-generation bioethanol (SGB) has emerged as an alternative to make use of the abundant, renewable, and inexpensive lignocellulosic biomass. Lignocellulosic biomass is composed of different polysaccharides, such as cellulose and hemicellulose, which can be a source of several fermentable sugars, of which xylose and glucose are the predominant sugars [4, 7]; in addition to several inhibitors derived from the transformation of sugars and lignin, from which acetate is present in a higher concentration than the others [8, 9]. After obtaining these syrups the efficient and fast utilization of pentose and hexose sugars, preferably simultaneously, is advantageous [10–14].

Traditionally, wild-type microorganisms have been used at industrial scale to produce FGB. However, these microorganisms are not able to efficiently ferment pentose sugars into SGB [10, 15–17], which is a significant disadvantage because pentose sugars may constitute up to 40% of the total sugars in the biomass [4, 7, 13, 18]. Due to these constraints, several microorganisms, such as yeasts and bacteria have been isolated or genetically modified, like *Escherichia coli*, *Saccharomyces cerevisiae* and *Zymomonas mobilis*, among others, to generate new biocatalysts capable to consume pentose and hexose sugar mixtures, for efficiently producing SGB [19–23]. For such purpose, *E. coli* shows several advantages, such as efficient consumption of hexoses and pentoses present in the hemicellulosic hydrolysates [24, 25] and high tolerance to various toxic compounds [9, 19, 22, 26]. Despite these advantages, *E. coli*, and other wild-type and metabolic engineered ethanologenic microorganisms, show the phenomenon of carbon catabolite repression (CCR), which is a regulatory system used by different microorganisms for the utilization of a preferred carbon source [7, 25, 27]. In the case of ethanologenic *E. coli* growing in mineral media with a mixture of sugars or biomass hydrolysates, glucose is always consumed first, and then arabinose, xylose, and other sugars are utilized after glucose depletion [7, 28, 29], causing a sequential utilization of mixed sugars, and often resulting in a delay and incomplete consumption of secondary sugars. This phenomenon limits ethanol titer, overall yield, and productivity [7, 13, 25, 28].

To overcome the phenomenon of CCR, strategies such as strain selection or metabolic engineering have been developed to engineer strains that can co-ferment mixtures of sugars simultaneously [7, 25, 28]. However, these strategies cause pleiotropic effects, such as significant reductions in the growth rate. An alternative to reduce or eliminate the effect of CCR, when a mixture of sugars is used in the culture medium, is the use of cell culture strategies such as continuous culture. Continuous cultures, besides being used as a tool to select novel strains through adaptive evolution, are also used for other purposes. For instance, it is used to maintain process variables in a steady state and perform omics analysis: fluxomic, transcriptomic or proteomic. For production purposes it is also utilized to increase and extend the volumetric and specific productivities, and yields of relevant metabolites [30–32]. In this regard, single- and multi-stage continuous cultures are widely used in the commercial production of FGB [15, 18, 21, 32–34], however, little has been reported regarding the use of continuous culture to ferment mixtures of sugars (pentoses and hexoses). Furthermore, continuous culture, as well as fed-batch cultures, has potential advantages over batch cultures as the specific growth rate can be controlled, a higher and long-term volumetric productivity can be obtained through manipulation of the feeding rate, and low concentrations of substrates and metabolic product inhibitors, can be maintained in the bioreactor to reduce inhibition of growth. In addition, continuous cultures offer reduced downtime for cleaning, filling, and sterilization, which can be translated into the use of smaller fermenters volumes and plant size, lower capital investments, and the reduction of production costs, once the steady-state is reached, compared to batch and fed-batch cultures [15, 18, 31, 32]. However, this culture method can also show some drawbacks such as: increased needs for process control and operation to optimize process conditions to reach the desired productivity; increased susceptibility to microbial contamination; the possibility of generating mutants or revertant strains (genetic instability) when the system is operated for a long time [15, 18, 34]; and the low concentration of ethanol resulting from a continuous process is particularly problematic given the cost for downstream processing, i.e. distillation.

This work aimed to evaluate the use of single- and two-stage continuous cultures under micro-aerated conditions on the simultaneous consumption of glucose and xylose, ethanol production, and volumetric ethanol productivity by the ethanologenic strain *E. coli* MS04. The experiments were performed in mineral medium supplemented with xylose, glucose, and acetate, simulating the composition of acid diluted biomass hydrolysates generated from the hemicellulosic fraction of lignocellulosic biomass. The results showed that two-stage continuous culture was better than batch and single-stage continuous culture regarding the volumetric ethanol productivity by ethanologenic *E. coli* MS04, promoting the simultaneous and total consumption of pentose and hexose sugars to potentially produce SGB.

## Methods

### Microorganism and media composition

The ethanologenic strain used in this study was *E. coli* strain MS04 (*E. coli* MG1655: *ΔpflB*, *ΔadhE*, *ΔfrdA*, *ΔldhA*, *ΔxylFGH*, *gatC S184L*, *Δreg 27.3kb*, P*pflB::pdc*_*Zm*_-*adhB*_*Zm*_) which was engineered to produce ethanol as the primary product [22]. The mineral medium used in this work was the modified AM1 mineral medium [22], which was used in batch, single- and two-stage continuous experiments carried out in this study.

### Inoculum preparation

Seed cultures of strain MS04 were prepared by inoculating 1.5 mL of a frozen stock (40 w/w glycerol solution and cells grown in AM1 medium) into 100 mL of AM1 medium supplemented with glucose (7.5 g/L), xylose (42.5 g/L) and sodium acetate (2 g/L) in a 500-mL baffled shake flask, which was incubated at 37 °C with an initial pH of 7.0 and 300 rpm. After 12 h of cultivation, cells were harvested by centrifugation (10,000×*g*, 10 min, 4 °C), and resuspended into the same mineral medium to start a batch culture, which had an initial inoculum equivalent to 0.1 optical density at 600 nm (OD_600_).

### Batch culture under micro-aerated conditions

Batch cultures were performed using a working volume of 750 mL, pH controlled at 7.0, 37 °C, 400 rpm, 0.1 vvm of air, corresponding to a volumetric oxygen transfer coefficient (*k*_L_a) of 7.2 h^−1^ [35]. Oxygen tension was measured using a sterilizable dissolved oxygen electrode (Ingold, model A420), as previously described [35]. Mineral medium AM1 [36] supplemented with glucose (7.5 g/L), xylose (42.5 g/L), and sodium acetate (2 g/L) was used in this study to simulate the composition of diluted hemicellulosic hydrolysates [37, 38]. The concentrations of sugars and acetate previously mentioned were also chosen to maintain a concentration of solutes where cells were not drastically inhibited by osmotic effects, since it is already known that bacteria can be inhibited by carbon sources at concentrations higher than 50 g/L. The mineral medium was prepared with sugars, and salts solutions sterilized separately by filtration and added to the bioreactor aseptically before inoculation. After 2 h of inoculation, the oxygen tension dropped to 0% of the air saturation.

### Single-stage continuous culture experiments under micro-aerated conditions

Single-stage continuous cultures (SSCC) were conducted by keeping the same medium composition and process variables as those used in batch culture experiments. SSCC were started as a batch culture, inoculated to an OD_600_ of 0.1, and were allowed to run for 12 h. Afterwards, the bioreactor was set up on continuous culture mode, by switching on a pre-calibrated peristaltic feed pump to supply sterile feed medium from the feed medium reservoir. To maintain a constant liquid culture volume (V = 750 mL), spent medium was continuously withdrawn at the same flow rate than the feed pump. Various feed flow rates (F; L/h) were tested, thus evaluating different dilution rates (D = F/V) [31], which are equal to the specific growth rate (D = µ; h^−1^) at steady state.

Five fixed D were evaluated in this culture mode: 0.05, 0.1, 0.15, 0.2 and 0.3 h^−1^. Steady-state was assumed when the cell, sugars, and ethanol concentrations did not change over time after three successive samplings, which was generally when the system ran for a period corresponding to 3–5 times the liquid residence time (θ_c_ = 1/D) [31]. Culture samples were analyzed immediately to determine cell, residual sugars, and ethanol concentrations.

### Two-stage continuous culture experiments under micro-aerated conditions

To evaluate the consumption of glucose and xylose, and the production and productivity of ethanol in multi-stage continuous cultivations, two-stage continuous cultures (TSCC) were performed. Two fermenters were connected in series with a working volume of 750 mL each (combined working volume of 1.5 L) and were fed with fresh mineral medium only in the first stage, thus evaluating the same D in both stages (D_1_ = D_2_). Medium composition and process variables were the same as single-stage continuous culture. The first stage of the TSCC was initiated as previously described with the single-stage continuous culture, but the effluent stream from this stage was sent to the subsequent second stage with a peristaltic pump, and then the spent medium from the second stage was transferred to the waste bottle using a third outlet pump. Four fixed dilution rates were evaluated in both stages (0.1, 0.15, 0.2 and 0.3 h^−1^). Again, the steady-state was assumed when cell, sugars and ethanol concentrations did not change over time in both stages (3–5 θ_c_). Unlike SSCC, the specific growth rate at the second stage (µ_2_) was always lower than the dilution rate at the same stage (D_2_) because of the entry of biomass in the second stage, coming from the first stage. The µ_2_ was calculated according to Eq. 1, which is derived from the mass balances on TSCC.

1 $$\mu_{2} = \frac{{D_{2} \left( {x_{2} - x_{1} } \right)}}{{x_{2} }}$$

where *x*_1_ and *x*_2_ are the steady-state cell concentrations at the first and second stage of TSCC, respectively.

### Analytical methods

Cell concentration was determined spectrophotometrically as optical density at 600 nm (DU-70, Beckman Instruments, Inc. Fullerton, CA), and converted to dry cell weight (DCW) per liter using a calibration curve (1 optical density at 600 nm = 0.37 g_DCW_/L). Glucose and xylose concentrations were measured with a biochemical analyzer (YSI model 2700, YSI Inc., Yellow Springs, OH); whereas acetate concentrations were measured by high-performance liquid chromatography [22]. Ethanol concentrations were measured by gas chromatography using *n*-butanol (1%) as an internal standard (6850 Series GC System, Agilent, Wilmington, DE, USA) as previously reported [22]. The volumetric ethanol productivity (Q_P_) in the SSCC and TSCC was calculated as the product of the operating dilution rate and ethanol concentration produced at the first (Eq. 2) and the second stage (Eq. 3), respectively. The specific ethanol productivity (q_P_) in each stage was calculated as the product of the specific growth rate (µ) and the specific product yield (Y_PX_) at the steady state (Eq. 4). Theoretical ethanol yields on sugars consumed (Y_PS_) in each stage in continuous culture, as well as in batch culture, were calculated as previously described [35], and are expressed as a percentage of the theoretical maximum yield (0.51 g_ethanol_/g_sugar_).

2 $$Q_{P1} = D_{1} P_{1}$$

3 $$Q_{P2} = D_{2} \left( {P_{2} - P_{1} } \right)$$

4 $$q_{P} = \mu Y_{PX}$$

## Results and discussion

### Batch culture with the ethanologenic strain *E. coli* MS04 under micro-aerated conditions displays sequential consumption of glucose and xylose

**Fig. 1:**
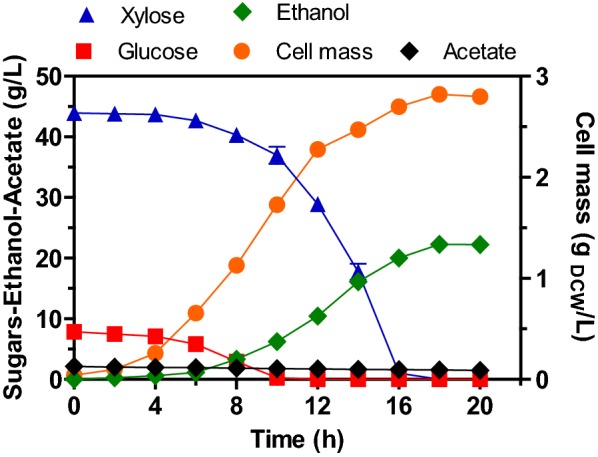
Growth, sugar consumption and acetate, and ethanol production kinetics by the engineered strain *E. coli* MS04 in micro-aerated batch culture using mineral medium supplemented with xylose, glucose and sodium acetate (42.5, 7.5 and 2 g/L, respectively)

Ethanologenic *E. coli* strain MS04 was previously engineered and evolved to produce ethanol as the primary fermentation product from hexose and pentose sugars in the presence of high concentrations of acetate [22]. As previously reported [22], compared to the absence of acetate, concentrations of this acid ranging from 2 to 10 g/L promote an increase in the specific growth rate, cell mass formation, and ethanol volumetric productivity. This is probably due to an increase in the synthesis of acetyl-CoA from acetate because the genes for its catabolism were not interrupted and the adaptive evolution process improved acetate tolerance in strain MS04 [22]. In batch culture, the total concentration of sugars (50 g/L) was consumed in 18 h, with an ethanol production of 22 g/L, and a cell mass concentration close to 3 g_DCW_/L (Fig. 1). The consumption of sugars at the beginning of the culture was semi-sequential, with glucose being consumed during the first 6 h, followed by a period of simultaneous consumption of glucose and xylose (from 6 to 10 h of culture), until depletion of the former and then xylose being depleted between 16 and 18 h. Kinetic parameters, such as µ and q_P_ at the exponential growth phase, as well as Y_PS_, Y_XS_, Q_P_ and carbon balance were estimated at the global phase and are shown in Table 1. A significant fraction of the carbon was directed towards the production of ethanol, with a minimal amount directed to synthesize cells or other by-products (Table 1). For instance, the cell yield on consumed sugars (Y_XS_) was only 0.054 g/g, there was a negligible production of acetate (Fig. 1), and a carbon recovery of 91% was obtained. About 25% of the acetate present initially in the medium was consumed, being this quantity probably used for biosynthetic pathways [22], whereas the residual concentration (1.5 g/L) remained constant throughout the culture.

**Table 1:** Kinetic and stoichiometric parameters of ethanologenic *E. coli* MS04 in batch and SSCC under micro-aerated conditions in mineral medium supplemented with xylose (42.5 g/L), glucose (7.5 g/L), and sodium acetate (2 g/L)

Culture	D (h^−1^)	μ (h^−1^)	Ethanol (g_EtOH_/L)	Y_PS_ (%, w/w)	Q_P_ (g_EtOH_/L h)	q_P_ (g_EtOH_/g_DCW_ h)	Y_XS_ (g_DCW_/g_S_)	Carbon balance (%)
Batch	–	0.46 ± 0.01	22.2 ± 0.09	87 ± 1.2	1.3 ± 0.05	1.38 ± 0.02	0.054 ± 0.001	91 ± 0.91
SSCC	0.05	0.05	18.2 ± 0.15	72 ± 0.6	0.9 ± 0.01	0.58 ± 0.03	0.032 ± 0.002	75 ± 0.55
SSCC	0.10	0.10	15 ± 0.4	82 ± 1	1.5 ± 0.04	0.81 ± 0.04	0.051 ± 0.002	91 ± 0.55
SSCC	0.15	0.15	10 ± 0.2	82 ± 2	1.5 ± 0.04	0.99 ± 0.02	0.064 ± 0.005	95 ± 1.3
SSCC	0.20	0.20	5.1 ± 0.2	77 ± 2	1.0 ± 0.04	1.04 ± 0.03	0.076 ± 0.003	96 ± 0.7
SSCC	0.30	0.30	1.8 ± 0.2	80 ± 2	0.53 ± 0.06	1.47 ± 0.02	0.087 ± 0.014	99 ± 0.5

Average values and standard errors are shown from duplicate experiments for batch cultures and triplicate measurements during the steady state of continuous cultures

*D* dilution rate, *µ* specific growth rate, *Y*_*PS*_ ethanol yield on sugars consumed as percentage of the maximum theoretical; *Y*_*XS*_ biomass yield on sugars consumed, *Q*_*P*_ volumetric ethanol productivity, *q*_*P*_ specific ethanol productivity

Ethanologenic *E. coli* MS04 showed a high µ (0.46 h^−1^) when it grew in the presence of a xylose/glucose mixture (50 g/L) in mineral medium added with sodium acetate, as well as a Y_PS_ close to 90% of the theoretical maximum, and a high Q_P_ and q_P_ (1.3 g/L h and 1.38 g/g h, respectively; Table 1). The kinetic and stoichiometric parameters of the strain MS04 are among the highest reported by ethanologenic microorganisms, such as strains of *E. coli* and *S. cerevisiae*, growing in batch culture on synthetic media supplemented with xylose or glucose/xylose mixtures [39–43]. Even though there are differences among the studies, such as the metabolic background of each strain, the culture media, the implementation of metabolic evolution processes, the process conditions (aerated or non-aerated) used in each case, among others [39–43]. In the case of strain MS04, the rate of xylose transport into the cell, and/or the metabolic flux to produce ethanol may be higher, and therefore the strain MS04 can metabolize xylose and produce ethanol at higher rates in comparison to other ethanologenic bacteria or yeast strains [39–43].

On the other hand, process conditions, such as agitation and aeration could also influence the productivity of the process. The main variable in this study was the oxygen transfer rate, which depends on the agitation and aeration. The aerated conditions used in this study (0.1 vvm, 400 rpm, *k*_L_a = 7.2 h^−1^) were previously described as optimal for the consumption of glucose/xylose mixtures, and production of ethanol by *E. coli* MS04 [35]. Therefore, high values of µ, q_P_, and Q_P_ (1.3 g/L h) were reached in this study compared to the parameters found using other ethanologenic strains of *E. coli* and *S. cerevisiae* metabolizing xylose/glucose mixtures (Q_P_ from 0.55 to 0.92 g/L h), where no air was supplied to the media, or even anaerobic conditions were maintained in the culture [39, 43–45].

Even though strain MS04 shows relevant advantages with respect to the production of ethanol from glucose/xylose mixtures in batch culture, the phenomenon of catabolite repression by glucose is still present, avoiding the co-fermentation of xylose until the concentration of glucose in the medium was lower than 6 g/L (Fig. 1), thus diminishing the volumetric ethanol productivity and the overall ethanol yield. Therefore, as proposed above, the use of continuous culture could provide a release from the catabolite repression, and thus increase the rate of consumption of sugars and ethanol productivity.

### Single-stage continuous culture under micro-aerated conditions promotes the total and simultaneous consumption of glucose and xylose at low specific growth rates

Continuous culture experiments were carried out to determine if this condition would promote the simultaneous consumption of sugars (glucose and xylose) and increase the volumetric productivity with a minimum effect on the ethanol yield compared to batch culture.

**Fig. 2:**
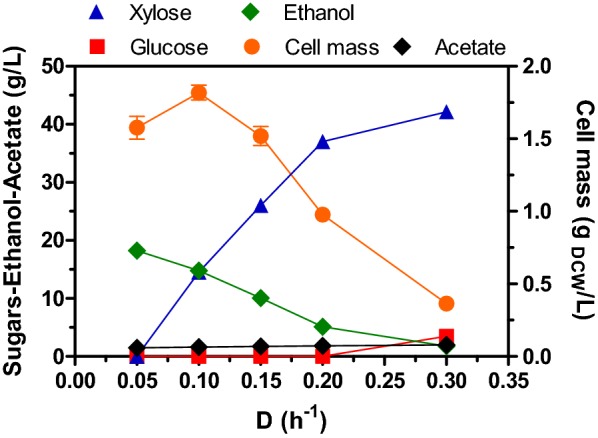
Cell mass, residual sugars and acetate, and ethanol concentration at steady-state of SSCC under micro-aerated conditions at different values of D by *E. coli* MS04. Feeding medium as described in Fig. 1

The results of cell mass, residual sugars, and ethanol concentrations by the strain MS04 at the steady-state of SSCC are shown in Fig. 2, where a complete consumption of the glucose/xylose mixture (7.5/42.5 g/L respectively) and the highest ethanol concentration (18.2 g/L) was obtained at the lowest D (0.05 h^−1^) tested (Table 1). Similarly to results found in the batch experiments, about 0.5 g/L of acetate was consumed at steady state of all D’s tested in SSCC (Fig. 2). As D increased, the simultaneous consumption of the glucose/xylose mixture was also observed, with the total consumption of glucose (7.5 g/L), and the consumption of xylose ranging from 5.5 to 28 g/L at D’s = 0.1–0.2 h^−1^. At D of 0.3 h^−1^, only a small concentration of glucose and xylose was consumed (4 and 0.4 g/L, respectively), and cell mass and ethanol production were reduced to 0.36 g_DCW_/L and 1.8 g/L, respectively (Fig. 2). At this D, approximately 3.5 g/L of glucose remained in the culture broth, slowing the xylose consumption substantially due to the catabolic repression effect exerted by glucose. Furthermore, the specific growth rate of strain MS04 cultured in similar conditions but using batch cultivations with xylose (50 g/L) as the sole carbon source was in the range of 0.21 to 0.25 h^−1^ (data not shown), i.e. 54–45% lower than that found with the glucose–xylose mixture in batch fermentations. However, at all dilutions tested, there was co-fermentation of glucose and xylose at different ratios in a single stage, which was influenced only by the dilution rate used in the experiments. In all cases, the concentration of cell mass was lower than that obtained in the batch cultures (< 2 g_DCW_/L), probably due to the dilution effect and the inhibitory effect of ethanol produced. Concerning the Q_P_, the highest value (1.5 g/L h) was reached at D = 0.1 and 0.15 h^−1^, which was higher than that obtained in batch culture (Table 1), though xylose was not totally consumed at these conditions (Fig. 2). The q_P_ had a direct relationship with D, and increased from 0.58 to 1.47 g/g h, in the range of D = 0.05–0.3 h^−1^ (Table 1). Furthermore, the Y_PS_ in SSCC was maintained practically constant (≈ 80%) in the range of D = 0.1–0.3 h^−1^, and only diminished to 70% when the D was 0.05 h^−1^ (Table 1). With respect to the Y_XS_ and carbon balance, it was observed that both parameters had also a direct relationship with D, showing that at low dilutions less carbon was directed to the synthesis of biomass, probably due to the redirection of substrate consumption for cellular maintenance processes, under the stress conditions derived from the high concentrations of ethanol (Table 1).

Comparing the results obtained in SSCC with respect to batch culture under the same process conditions, SSCC was superior in terms of volumetric and specific ethanol productivities, which increased approximately 20% and 7%, at D = 0.1–0.15 h^−1^, and D = 0.3 h^−1^, respectively (Table 1). The opposite was observed for the case of titer and yield, where these parameters were lower, at all conditions tested, to those obtained in batch culture (Table 1). This kind of behavior is commonly observed in SSCC, where higher Q_P_ and q_P_ are reached at an optimum D; while titer, yield and, the carbon balance are lower at low dilutions in comparison to batch culture [15, 18, 44, 46]. The reduction in the overall ethanol yield and carbon balance at D = 0.05 h^−1^, but with the higher concentration of ethanol achieved (18 g/L) (Table 1), suggest that some carbon was lost as CO_2_. Finally, because of the micro-aerated conditions used in this study (*k*_L_a = 7.2 h^−1^), it is also possible that a certain amount of CO_2_ was released from the microaerobic metabolism in the tricarboxylic acid cycle, which was not quantified at the exit of the fermentor. In this respect, this behavior has also been observed with other ethanologenic *E. coli* strains, such as ATCC 11303 and FBR5 (both transformed with the plasmid pLOI297), which were grown in batch and SSCC, and both produced lower ethanol titer and yield in SSCC compared to the values reached in batch culture under the same conditions [18, 44]. Lower ethanol yields in SSCC were also obtained [46] (about 90% of the theoretical) when two immobilized ethanologenic *E. coli* strains (AFF01 and CT1101) were co-cultured to convert glucose/xylose mixtures to ethanol, compared to batch culture, where a yield of more than 95% was achieved [46]. The authors proposed that the lower yield in SSCC was due to the continuous exposure of immobilized cells to high concentrations of ethanol, unlike when the cells were grown in batch culture, where the exposition to high ethanol concentrations was only present at the end of the culture when the sugars were exhausted. They also suggested that the incomplete utilization of sugars in continuous culture by the immobilized cells was because of the inhibition of ethanol [46]. Thus, even with the possible inhibition of ethanol on growth and ethanol production by MS04, the performance of strain MS04 simultaneously fermenting mixed sugars to ethanol in SSCC with mineral medium is among the best reported so far, considering the sugar conversion, and the specific and volumetric productivities reached using mineral or complex media by other ethanologenic bacteria and yeast strains [18, 33, 44–50].

### Two-stage continuous culture promotes the total and simultaneous consumption of sugars allowing to achieve a high volumetric ethanol productivity

**Fig. 3:**
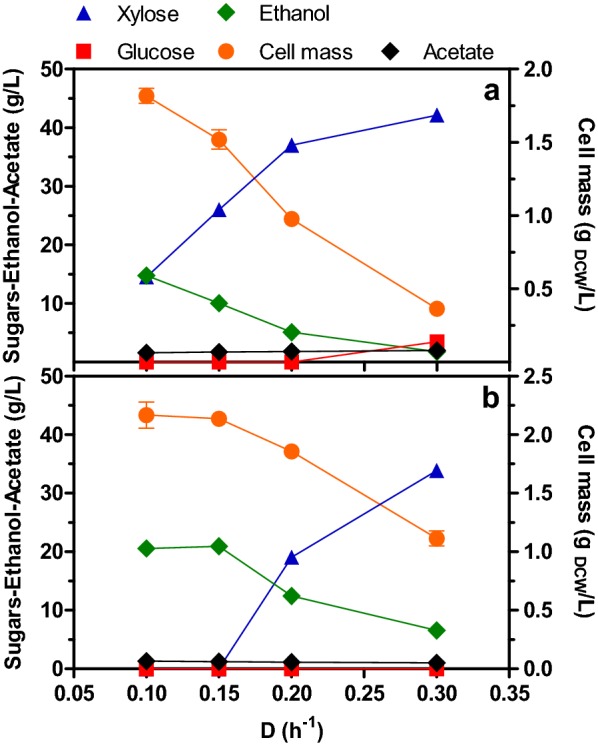
**a** Steady-state concentration of cell mass, residual sugars and acetate, and ethanol by *E. coli* MS04 at the first stage of a micro-aerated TSCC. **b.** Steady-state concentration of cell mass, residual sugars and acetate, and ethanol by *E. coli* MS04 at the second stage of a micro-aerated TSCC. Medium was fed at the first stage as described in Fig. 1

To determine if it would be possible to maintain high volumetric ethanol productivities in continuous culture with a higher consumption of sugars and production of ethanol in the system, two-stage continuous cultures (TSCC), under micro-aerated conditions (*k*_L_a = 7.2 h^−1^), were performed to consume the residual sugars exiting the SSCC when D was > 0.05 h^−1^. The working volume and D were the same in both stages of the TSCC. Four values of D were tested (0.1, 0.15, 0.2 and 0.3 h^−1^), feeding AM1 mineral medium supplemented with xylose, glucose and sodium acetate (42.5, 7.5 and 2 g/L respectively) only to the first stage. Figure 3a, b show the concentrations, at the steady state, of cell mass, residual sugars (xylose and glucose), acetate and ethanol at the first and second stage, respectively, in the TSCC at different values of D. As mentioned before, the first stage of TSCC had the same behavior as SSCC, with the presence of residual sugars in the effluent at D_1_ ≥ 0.1 h^−1^, and an inverse relationship between D_1_ and cell mass, ethanol production, consumed sugars and acetate (Fig. 3a). The highest value of Q_P_ (~ 1.5 g/L h) was attained at D_1_ of 0.1 h^−1^ and 0.15 h^−1^ and decreased as D_1_ increased (Table 2). The highest Y_PS_ was 82% at a D_1_ of 0.15 h^−1^ and decreased to about 80% at the other D_1_′s tested (Table 2).

**Table 2:** Kinetic and stoichiometric parameters of the strain *E. coli* MS04 calculated at both stages in TSCC, and as a global TSCC (G) under micro-aerated conditions in mineral medium supplemented with xylose (42.5 g/L), glucose (7.5 g/L) and sodium acetate (2 g/L)

μ_1_ (h^−1^)	μ_2_ (h^−1^)	μ_G_ (h^−1^)	Q_P1_ (g_EtOH_/L h)	Y_PS1_ (%)	Q_P2_ (g_EtOH_/L h)	Y_PS2_ (%)	Q_PG_ (g_EtOH_/L h)	Y_PSG_ (%)
0.10	0.018	0.050	1.48 ± 0.04	81 ± 1.0	0.58 ± 0.04	78 ± 2.5	1.0 ± 0.01	81 ± 0.5
0.15	0.043	0.075	1.51 ± 0.04	82 ± 0.8	1.63 ± 0.07	82 ± 1.5	1.6 ± 0.03	82 ± 0.9
0.20	0.095	0.100	1.00 ± 0.04	77 ± 3.0	1.46 ± 0.09	81 ± 3.0	1.2 ± 0.05	79 ± 3.0
0.30	0.200	0.150	0.53 ± 0.06	80 ± 3.5	1.44 ± 0.07	80 ± 3.5	1.0 ± 0.05	80 ± 3.0

Average values and standard errors are shown from triplicates

μ_**1**_, μ_**2**_, μ_**G**_: specific growth rates at stage 1 and 2 of the two-stage continuous culture, and at the continuous global culture, respectively

Q_P1_, Q_P 2_, Q_PG_: ethanol volumetric productivity at stage 1 and 2 of the two-stage continuous culture, and at the continuous global culture, respectively

Y_PS1_, Y_PS2_, Y_PSG_: ethanol overall yield at stage 1 and 2 of the two-stage continuous culture, and at the continuous global culture, respectively

G: continuous global culture was considered as a single-stage continuous culture with the sum of volumes of both stages in the two-stage continuous culture and fed at the same flow rate. D_G_ = D_1_/2 = D_2_/2

For the second stage of TSCC, operated at the same D as the first stage, at a D_2_ of 0.1 and 0.15 h^−1^, the remaining xylose concentration leaving the first stage (15 and 29 g/L, respectively) was completely consumed, and only when the system was operated at D_2_ ≥ 0.2 h^−1^, xylose was present in the effluent medium up to a concentration of 34 g/L (Fig. 3b). The maximum concentration of ethanol reached at the second stage was 21 g/L at D_2_ = 0.1 and 0.15 h^−1^, and decreased to 7 g/L as D_2_ increased to 0.3 h^−1^ (Fig. 3b). Maximum cell mass concentration of 2.2 g_DCW_/L was attained at the lowest D_2_ (0.1 h^−1^), which was lower than the concentration reached in batch culture (2.8 g_DCW_/L), and it also had an inverse relationship with D_2_, as in the case of the first stage (Fig. 3b).

On the other hand, the concentration of acetate consumed in the second stage was similar to that consumed in the first stage (~ 0.5 g/L). At the exit of the second stage, there was a residual concentration of acetate ≥ 1 g/L, therefore, the acetate present in the feed medium was not completely consumed in the second stage. As previously reported [22], we suggest that low amounts of acetate are necessary for the synthesis of acetyl-CoA and other biosynthetic molecules. Q_P_ reached a maximum of 1.6 g/L h at D_2_ = 0.15 h^−1^, followed by the conditions when D_2_ ≥ 0.2 h^−1^, with the lowest value (0.6 g/L h) obtained at D_2_ = 0.1 h^−1^ (Table 2). In turn, values of Y_PS_ in the second stage were in the range of 78–82% of the theoretical for D_2_ ≥ 0.1 h^−1^ (Table 2).

With the aim of evaluating the TSCC as a system, and not only as two fermenters connected in series, the process parameters of TSCC were estimated as a continuous global culture, where both stages are fused into a single stage continuous culture with the sum of working volumes of both stages (1.5 L) and fed at the same flow rate as each D was operated in TSCC. Under these conditions, the dilution rate of the continuous global culture (D_G_) was half of D_1_ or D_2_ in TSCC, and at the same time, D_G_ was equal to the specific growth rate in the global continuous culture (µ_G_) (Table 2). The residual xylose and ethanol concentration in the global steady-state were the same as those in the steady-state of second-stage of TSCC, with the highest residual xylose concentration (34 g/L) at D_G_ = 0.15 h^−1^ (D_1_ = D_2_ = 0.3 h^−1^), and a maximum concentration of ethanol of 21 g/L at D_G_ = 0.05 and 0.075 h^−1^. With these data, process parameters were calculated and are presented in Table 2, with a maximum Y_PS_ of 82% at a D_G_ of 0.075 h^−1^, and about 80% of the theoretical in the other D_G_′s. The maximum Q_P_ achieved (1.6 g_EtOH_/L h) was also obtained at a D_G_ of 0.075 h^−1^, with an increase of 23% and 78% compared to batch culture and SSCC, respectively, and it diminished to 1.0 g_EtOH_/L h at a D of 0.3 h^−1^ (Table 2).

The advantages of using multi-stage continuous culture (MSCC) over batch and SSCC with respect to volumetric productivity, product concentration, and substrate utilization, have been documented elsewhere [15, 32, 34]. One of the main advantages of using MSCC instead of batch culture or SSCC is the feasibility of operating each stage separately and independently, with different process conditions, to find the optimal conditions in each stage to reach the highest substrate conversion, product concentration and productivity in the system [32]. Thus, when comparing a SSCC with an equivalent MSCC, both operated at the same flow rate, total working volume, and global D, the later will show better results regarding product concentration and productivity, thus improving the technical and economic feasibility of the process [15, 34].

In this study, we used the same D and working volume in each stage of the TSCC to ferment a mixture of sugars (50 g/L), consisting of glucose and xylose, achieving the total conversion of sugars, and reaching a Q_P_ of 1.6 g/L h at a D_G_ of 0.075 h^−1^. These results are promising when considering the use of the mineral medium, the absence of antibiotics in the medium, and the use of a high xylose/glucose ratio, in comparison to other results where complex media, the presence of antibiotics, and/or low xylose concentrations are used. Some authors have reported the use of TSCC to ferment mixed sugars to ethanol with ethanologenic yeast, bacteria, or by using both in a co-culture, inoculating each in different stages. For instance, two recombinant *S. cerevisiae* strains, LNH33 and LNH-ST, were separately cultivated in TSCC, with complex medium supplemented with xylose (34 g/L) and glucose (24 g/L), and a working volume of 1 L each, at D of 0.042–0.043 h^−1^ in both stages, under non-aerated conditions [49, 50]. The results with both strains (LNH33 and LNH-ST) showed that glucose was completely consumed in the first stage, but xylose was partially converted (11.4 and 58.3% conversion, respectively) [49]. At the steady stage of the second stage xylose conversion reached a maximum of only 86.4%, with an ethanol production of 13.8 and 21 g/L, respectively [49].

In this case, both strains were unable to convert the total concentration of xylose in the feed, even with the use of two-stages connected in series at a low dilution rate [49]. Another study also reported the co-fermentation of mixed sugars (glucose, 30 g/L; xylose, 15 g/L) with the recombinant *S. cerevisiae* strain 424A (LNH-ST), without aeration, in MSCC on YPD complex medium, and with three reactors of different working volumes connected in series [50]. At the steady-state, all glucose and 37% of xylose (5.6 g/L) were consumed in the first stage (D = 0.05 h^−1^); however, with the use of the other two stages, only a conversion of xylose of 69% was reached at the exit of the third stage (D = 0.05 h^−1^) [50].

Lastly, a combination of *Z. mobilis* and *Scheffersomyces stipitis* strains were also used to evaluate the conversion of sugar mixtures in TSCC [51]. The hexose-fermenting bacterium *Z. mobilis* strain MTCC91 was inoculated in the first stage, with no aeration; and the pentose-fermenting yeast *Scheffersomyces stipitis* strain CBS6054 was inoculated in the second stage under micro-aerated conditions (0.2 vvm). After testing different flow rates of complex medium supplemented with glucose, 80 g/L; and xylose, 40 g/L, it was found that the best condition was D_1_ = 0.071 h^−1^ and D_2_ = 0.048 h^−1^. At these dilution rates, the first stage allowed a glucose conversion of 81%, with no xylose conversion; while in the second stage, the remaining glucose was completely consumed, but only 62.5% of xylose was utilized by the yeast. The overall ethanol production was 50 g/L, equivalent to a Q_P_ of 1.56 g/L h [51]. The Q_P_ reported in that study was like the value obtained by MS04 strain in this study. However, the xylose/glucose ratio used by MS04 was higher, and the experiments were carried out using mineral medium, instead of complex medium.

In the present study, it is shown that TSCC was superior to SSCC since the total consumption of the sugar mixture was achieved at higher dilution rates (D_1_ = D_2_ = 0.15 h^−1^), in comparison to the required in SSCC (D = 0.05 h^−1^). The use of this higher D in TSCC allowed the system to reach a higher ethanol concentration and productivity at a given value of D in the steady state (Table 2), by simultaneously consuming the total concentration of sugars and a partial consumption of acetate, which was required only in small amounts for biosynthetic pathways. It is important to mention that the deletion of the gene encoding the native alcohol dehydrogenase (*adhE*) in strain MS04 makes unfeasible to metabolize acetate to ethanol via acetyl-CoA.: i.e. acetate → acetyl-CoA → ethanol. Furthermore, the conversion of acetate to pyruvate or phosphoenolpyruvate, also via acetyl-CoA, and then to ethanol by the recombinant ethanol pathway from *Z. mobilis* is also unlikely as acetyl-CoA should be metabolized through the tricarboxylic acid cycle. However, this metabolic route does not operate as a cycle under the limiting oxygen conditions tested. Hence, it is improbable that consumed acetate contributes to the formation of ethanol in the strain used in this study. The configuration of TSCC was also more efficient than batch culture because it showed a higher productivity and simultaneous consumption of sugars, as well as an operation that can be maintained for a long time, eliminating the downtime required in batch and fed-batch cultures, and thus extending the productivity of the system. As mentioned before, MSCC presents the advantages of operating each stage independently, thus it allows the evaluation of a different number of stages, working volumes, flow rates, media composition and operating conditions in each stage. The unlimited possibilities of operation in this type of systems make MSCC very attractive at the laboratory and industrial level. Also, MSCC could be used at production level with hemicellulosic hydrolysates, because these syrups usually have low viscosity and low amounts of suspended solids, which make them easy to pump to feed large-scale continuous fermenters, and, as shown here, can be managed to reduce the carbon catabolite repression phenomena and perform the co-fermentation of mixed sugars to produce SGB with ethanologenic *E. coli.*

As described above, all xylose was not consumed in stage one or in SSCC at D ≥ 0.1 h^−1^, and all glucose is not consumed at D_1_ = 0.3 h^−1^. As previously shown [35], at oxygen transfer rates above 1.55 mmol/(L h) (corresponding to k_L_a = 7.2 h^−1^ and dissolved oxygen values of zero) the specific growth rates and cell mass increased in batch cultures. Owing to the characteristics of chemostats, these facts suggest that, under the conditions evaluated in this study, cells in SSCC and TSCC were not carbon growth limited, but were oxygen limited for growth in stage 1. Higher k_L_a values to 7.2 h^−1^ were not tested because a value of 13.6 h^−1^ (i.e. 90% above the value used) in batch cultures [35] provoked an increase in growth rate and cell mass formation, but a significant decrease in ethanol titer and yield. As seen in Fig. 2, the second stage in the TSCC basically is a xylose-conversion chemostat to ethanol at D = 0.10–0.20 h^−1^, but not at dilution rates above 0.2 h^−1^. The growth rate of strain MS04 in batch cultures using glucose-xylose mixtures and a k_L_a of 7.2 h^−1^ is in the range of 0.46–0.47 h^−1^ [35; and this study]. However, on xylose this parameter is in the range of 0.20–0.25 h^−1^ (also at a k_L_a = 7.2 h^−1^; unpublished results). Hence, at D ≥ 0.2 h^−1^ the steady-state cell concentration in stage 2 was lower in comparison to lower dilutions, and not all xylose was consumed, and the ethanol titer decreased (Fig. 2). Moreover, cells did not wash-out because they were supplied from stage 1. Taking together these facts, probably in the second stage remnant xylose is efficiently metabolized to ethanol because, in comparison to SSCC, more oxygen is supplied, and the dilution rate is below the µ of strain MS04 grown on xylose as carbon source.

## Conclusions

The knowledge of used production kinetics and bioengineering techniques to maximize the simultaneous substrate utilization of media containing mixed sugars, and to increase the productivity of a specific metabolite, is of utmost importance in the conversion of lignocellulosic materials to biofuels. In this work, evolved *E. coli* strain MS04 showed a good performance in the conversion of xylose/glucose mixtures into ethanol, compared to other ethanologenic strains, when it grew in batch, single- and two-stage continuous cultures, achieving volumetric productivities higher than 1.5 g/L h in mineral media without antibiotics, and in the presence of inhibitors, such as acetate. It was demonstrated that the two-stage continuous culture is a better strategy than batch culture to co-ferment xylose/glucose mixtures to ethanol, since the ethanol productivity was higher in this system. In addition, for the conversion of higher concentration of xylose or mixed sugars, and the corresponding high ethanol productivities, the use of other configurations in continuous cultures, such as immobilization of the cells, or cell recycling (internal or external), could be a good alternative to enhance pentose sugars conversion, overall yield, and productivity of ethanol. The present work can be useful for further studies in continuous culture with recombinant ethanologenic strains, which could increase the simultaneous conversion of mixed sugars, thus eliminating or reducing the phenomenon of catabolite repression by glucose and enhancing the productivity of the system.

## Data Availability

All data generated or analyzed during this study are included in this published article.

## Abbreviations

*adh*: alcohol dehydrogenase gene
CCR: carbon catabolite repression
D: dilution rate
D_1_: dilution rate in the first-stage of two-stage continuous culture
D_2_: dilution rate in the second-stage of two-stage continuous culture
D_G_: global dilution rate in the two-stage continuous culture
DCW: dry cell weight
EtOH: ethanol
F: flow of feed medium
*k*_L_a: volumetric oxygen transfer coefficient
FGB: first-generation bioethanol
G: glucose
MSCC: multi-stage continuous culture
mM: millimolar
OD_600_: optical density at 600 nm
*pdc*: pyruvate decarboxylase gene
Q_P_: volumetric ethanol productivity
q_P_: specific ethanol productivity
SGB: second-generation bioethanol
SSCC: single-stage continuous culture
TSCC: two-stage continuous culture
V: working volume
X: xylose
Y_PS_: ethanol yield on sugars consumed as percentage of the maximum theoretical
Y_PX_: specific ethanol product yield
Y_XS_: cell yield on consumed sugars
x_1_: cell concentration at the steady-state of the first-stage of two-stage continuous culture
x_2_: cell concentration at the steady-state of the second-stage of two-stage continuous culture
µ: specific growth rate
µ_1_: specific growth rate in the first-stage of two-stage continuous culture
µ_2_: specific growth rate in the second-stage of two-stage continuous culture
µ_G_: global specific growth rate in the two-stage continuous culture
µ_max_: maximum specific growth rate
θ_c_: liquid residence time
